# The deubiquitinating enzymes-related signature predicts the prognosis and immunotherapy response in breast cancer

**DOI:** 10.18632/aging.206010

**Published:** 2024-07-09

**Authors:** Youyuan Deng, Jingyong Li, Ye He, Dou Du, Zhiya Hu, Chao Zhang, Qishuo Rao, Yiping Xu, Jianguo Wang, Ke Xu

**Affiliations:** 1 Department of General Surgery, Xiangtan Central Hospital, Xiangtan 410000, Hunan, P.R. China; 2 Department of Pathology, Xiangtan Central Hospital, Xiangtan 410000, Hunan, P.R. China; 3 Department of Pharmacy, The Third Hospital of Changsha, Changsha 410000, Hunan, P.R. China; 4 Department of Oncology, The First Affiliated Hospital of Chengdu Medical College, Chengdu 610500, Sichuan, P.R. China; 5 Clinical Medical College, Chengdu Medical College, Chengdu 610500, Sichuan, P.R. China

**Keywords:** breast cancer, deubiquitinating enzyme, prognosis, immunotherapy efficacy, OTUB2

## Abstract

Background: Breast cancer is a prevalent disease that has a dismal prognosis for patients and a bad outlook for treatments. Ubiquitination is a reversible biological process that regulates protein production and degradation, as well as plays a vital role in protein transport, localization, and biological activity.

Methods: We obtained the breast cancer patient sample data and used a machine learning technique to create a novel index called Deubiquitinating enzyme related index (DUBRI) by gathering genes associated to deubiquitinating enzymes. Based on DUBRI, we systematically analyze patients’ prognosis, clinical characteristics, tumor immune microenvironment, chemotherapy response and immunotherapy response. Finally, the function of OTUB2 was explored in breast cancer cells.

Results: DUBRI, which consists of five deubiquitinating enzyme genes (OTUB2, USP41, MINDY2, YOD1, and PSMD7), is a reliable predictor of survival in breast cancer patients. We found that the high DUBRI group presented higher levels of immune cell infiltration. We performed molecular docking prediction of core target proteins in deubiquitinating enzymes. *In vitro* experiments verified that knockdown of OTUB2 could inhibit the proliferation and migration of breast cancer.

Conclusions: The DUBRI discovered in this research may effectively evaluate the outlook of breast cancer patients and identify groups of patients who would gain advantages from immunotherapy, offering vital knowledge for the future targeted treatment of breast cancer patients.

## INTRODUCTION

Breast cancer is a common and fatal malignant tumor with 3,000,000 new cases and 685,000 deaths globally in 2020 according to data from the latest studies [1, 2]. Surgery, chemotherapy is the current mainstay of treatment for breast cancer. Despite the substantial progress made in the current treatment of breast cancer, its high heterogeneity and drug resistance result in advanced patients with high mortality rates [3]. Hence, it is essential to find personalized biomarkers and customize focused therapeutic approaches to enhance the results of breast cancer patients undergoing first clinical treatment.

Ubiquitination is a reversible biological process that regulates protein production and degradation while also influencing protein transport, location, and biological activity. Deubiquitinating enzymes (DUB) mediate the removal of ubiquitination modifications by shearing ubiquitin from substrate proteins, altering peptidases, and compiling ubiquitin chains [4] Current research has found that the ubiquitin-proteasome system influences a wide range of diseases, including cancer [5].

Machine learning algorithms can be used to customize and optimize diagnostic, therapeutic, and targeted treatment regimens for a variety of diseases, including cancer, by analyzing patient data from clinical trials [6]. Deubiquitinating enzymes have a significant role in the formation of malignant tumors and the resistance to drugs. Nevertheless, the precise nature of the connection between deubiquitinating enzymes and breast cancer has yet to be fully understood. This research introduced a novel metric called the deubiquitinating enzyme-related index (DUBRI). The DUBRI was developed by gathering genes associated with deubiquitinating enzymes and used machine learning techniques to forecast the prognosis and efficacy of treatment interventions in patients with breast cancer. We performed *in vitro* experimental assays to evaluate the role of OTUB2 in breast cancer progression.

## MATERIALS AND METHODS

### Data sources

We extracted transcriptomic data and clinical data from the TCGA database [7]. Patients with incomplete information or unknown survival status were excluded. We collected key genes expressing deubiquitinating enzymes as DUB-associated genes from the GSEA gene set, KEGG, Hallmark, and review articles (Supplementary Table 1). In addition, we obtained expression data and clinical characteristics of breast cancer from the GEO database (ID: GSE20685 and GSE88770) as a validation cohort (Supplementary Table 2).

### Construction and validation of deubiquitinating enzyme-related index (DUBRI)

The TCGA samples were used as the training dataset and the GEO dataset was merged and then used as the validation dataset. The training dataset was used to construct DUBRI-related features to predict the prognosis of breast cancer patients. The validation dataset was validated against DUBRI according to the formula of the training dataset. Afterwards, five DUB-related genes with the best prognostic values were obtained and DUBRI was constructed by uni-Cox regression analysis, last LASSO analysis and multi-Cox analysis.

### Gene set enrichment analysis (GSEA)

We acquired reference genomes from the MSigDB Database. Screening conditions were |NES| > 1, NOM p-value (p-value) < 0.05.

### Immune microenvironment analysis

The CIBERSORT method (https://cibersort.stanford.edu/) was used to determine the fraction of 22 different kinds of immune infiltrating cells. The ssGSEA technique was used to measure the relative number of immune cells invaded. Immune cell correlation analysis was conducted using XCELL, QUANTISEQ, MCPCOUNTER, EPIC, and CIBERSORT-ABS software.

### Chemotherapy response and immunotherapy response

We obtained gene expression data of cancer cells from the Tumor Pharmacovigilance Multi-Organics (GDSC) database [8] and determined IC50 values to evaluate the efficacy of chemotherapeutic medicines in patients. The TIDE algorithm is capable of deducing the effectiveness of immunotherapy in patients [9]. We downloaded anti-PD-1 and anti-CTLA4 IPS scoring data from the TCIA database [10].

### Molecular docking simulation

We used the Schrödinger program to conduct a screening of small-molecule medicines that have an affinity for certain target proteins. Additionally, we performed molecular docking simulations. We obtained the protein structure of the specified target (OTUB2-4FJV) from the PDB database and gathered naturally occurring small molecule medicines from the PubChem database. The Glide module of Schrödinger program was used to mimic the binding postures of OTUB2 with small molecule medicines.

### Bioinformatics analysis

Differential analysis of breast cancer and normal tissue was performed using the R package “limma”, with cut-off values set at log2 fold change (logFC) > 1.5 and adj p-value < 0.05 [11]. Heat maps were visualized using the R package “pheatmap” (23075208). Column plots and calibration curves were plotted using the R packages “rms” and “regplot”.

### Preparation of cell lines and lentivirus infection

The breast cancer cell lines MCF-7 and YCCB1 were purchased from ATCC, and the above cell lines have been verified and stored in the laboratory liquid nitrogen tank. The negative (shNC) and lentivirus-delivered shRNA-1 and shRNA-2 against OTUB2 (shOTUB2) were synthesized by GeneChem Corporation (Shanghai, China). The target sequences for shOTUB2 were as follows: 5’-CATCCCACTACAACATCCTTT-3’ (sh-OTUB2-1) and 5’-CGAGATGGATACCGCCCTGAA-3’ (sh-OTUB2-2).

### RNA extraction and quantitative PCR

RNA was extracted by TRIzol method, and the concentration and purity of RNA were measured. Reverse transcription kit (Tiangen, KR116-02) was used to reverse transcribe into cDNA. Quantitative PCR detection was performed by SYBR-dye method (Bio-Rad, 1725272), and a 10 μl reaction system was established for on-board detection of the tested samples. The qRT-PCR primers for OTUB2 were synthesized by Sangon Biotech: forward, 5’-TTCTTCGGGACCATCCTGAAA-3’, reverse, 5’-CCAGGTAGGAATAGCCCAAGG-3’.

### Western blot

Primary antibody incubation was carried out after gel dispensing, sample loading, electrophoresis, membrane transfer, and blocking. According to the instructions of OTUB2 antibody (Proteintech, 12066-1-AP), the primary antibody was diluted, the PVDF membrane was put in and incubated overnight, and then incubated by the second antibody. After washing the membrane, exposure was performed and the results were recorded.

### Cell function experiment

In this study, CCK8 assay, cell clone formation, Transwell experiment, and cell cycle detection were performed. CCK8 experiment: the cell concentration was adjusted to 2000/100 μl, and the cells were added to the 96-well plate. 4 hours later, the cells were attached to the wall, and CCK8 was added, incubated at 37° C for 1 hour, and the absorbance was detected by enzyme-linked immunosorbent assay (ELISA) reader. Cell clone formation: the number of inoculated cells was adjusted according to cell proliferation ability, and the cells were inoculated into the 6-well plate according to gradient. Culture for 10-14 days. When the clone grows to about 50 cells, the formation of the clone can be seen. Stop the culture, and then fix, stain and count. Transwell experiment: after starving the treated cells overnight, the cell concentration was adjusted, the Transwell chamber was placed into the culture medium containing 10% fetal bovine serum, and cell suspension was added to the upper chamber of the Transwell chamber. After 36-48 hours, the chamber was fixed, stained, and photographed with a microscope to complete the recording. Cell cycle detection: 75% ethanol fixed the cells overnight at 4° C, following the instructions of the cell cycle detection kit (KeyGen Biotech, KGA512), added RNA enzyme and PI, and stayed in dark for 30 minutes, and then detected by flow cytometry (Novocyte).

### Statistical analysis

The Kaplan-Meier technique was used to generate survival curves for the purpose of comparing the survival disparities between the two groups. p-value <0.05 was deemed to be statistically significant.

### Data availability statement

All data utilized in this study are included in this article and all data supporting the findings of this study are available on reasonable request from the corresponding author.

## RESULTS

### Construction of DUBRI in breast cancer

We identified 5 deubiquitinating enzymes related genes and constructed DUBRI by uni-Cox regression, LASSO and multi-Cox regression analyses. Our model derived the Deubiquitinating enzyme related index (DUBRI) for each patient by the following formula. DUBRI = (- 0.3836*OTUB2 exp.) + (0.8808*USP41 exp.) + (0.0592*MINDY2 exp.) + (0.2029*YOD1 exp.) + (0.0768*PSMD7 exp.).

### Validation and clinical relevance of DUBRI

**Figure 1:**
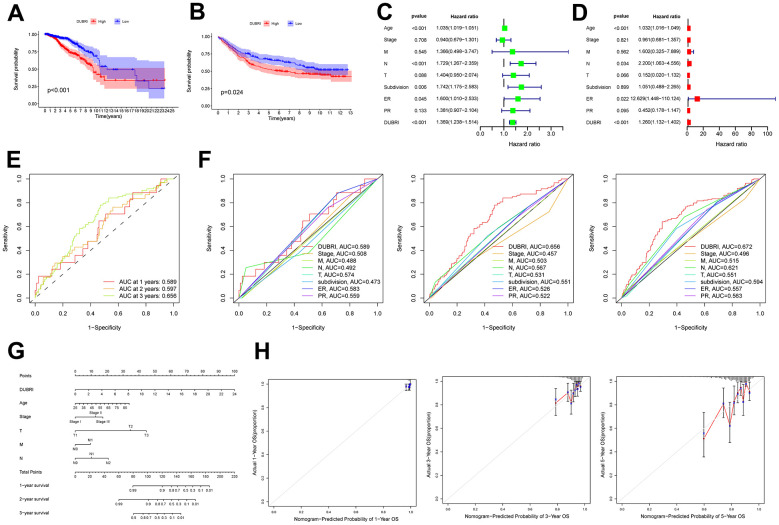
**External dataset validation and clinical correlation of DUBRI.** The Kaplan-Meier survival curves were used to compare the high and low DUBRI groups in both the TCGA dataset (**A**) and the GEO external dataset (**B**). Univariate (**C**) and multivariate (**D**) Cox regression analysis was used to examine the relationship between DUBRI and other clinical characteristics. (**E**) Receiver Operating Characteristic (ROC) curves depicting the performance of DUBRI at 1, 3, and 5 years. (**F**) Receiver Operating Characteristic (ROC) curves comparing the performance of DUBRI with various clinical features at 1, 3, and 5 years. (**G**) Prognostic column line graphs for breast cancer patients. (**H**) The provided data present calibration curves depicting the likelihood of overall survival at 1, 3, and 5 years in the TCGA cohort.

We discovered that patients in the high DUBRI group had a worse prognosis and were more likely to have a higher death rate (p < 0.05, Figure 1A). Patients with breast cancer who had higher DUBRI were more likely to have worse overall survival from GEO dataset (Figure 1B). In addition, we downloaded and analyzed the effectiveness of DUBRI in predicting patient prognosis in the external validation data METABRIC, and found that patients in the low DUBRI group had a worse prognosis compared to those in the high DUBRI group (Supplementary Figure 1).

Univariate and multivariate Cox regression analyses were performed to determine whether DUBRI could serve as an independent prognostic factor in breast cancer patients (Figure 1C, 1D). DUBRI had high accuracy in predicting 1-, 3-, and 5-year survival of breast cancer patients and was a better predictor of patient survival than other clinical traits (Figure 1E, 1F). Next, we used age, clinical stage, T, N, and M in the model to create column-line plots for breast cancer patients in order to evaluate their prognosis (Figure 1G). The calibrated column-line diagrams’ calibration curves revealed that the estimated 1-, 3-, and 5-year survival rates were more in line with the reference line’s actual survival rates. This suggests that the column-line diagrams that were created could accurately forecast the prognosis of individual patients (Figure 1H).

### DUBRI-based tumor microenvironment dissection in breast cancer

**Figure 2:**
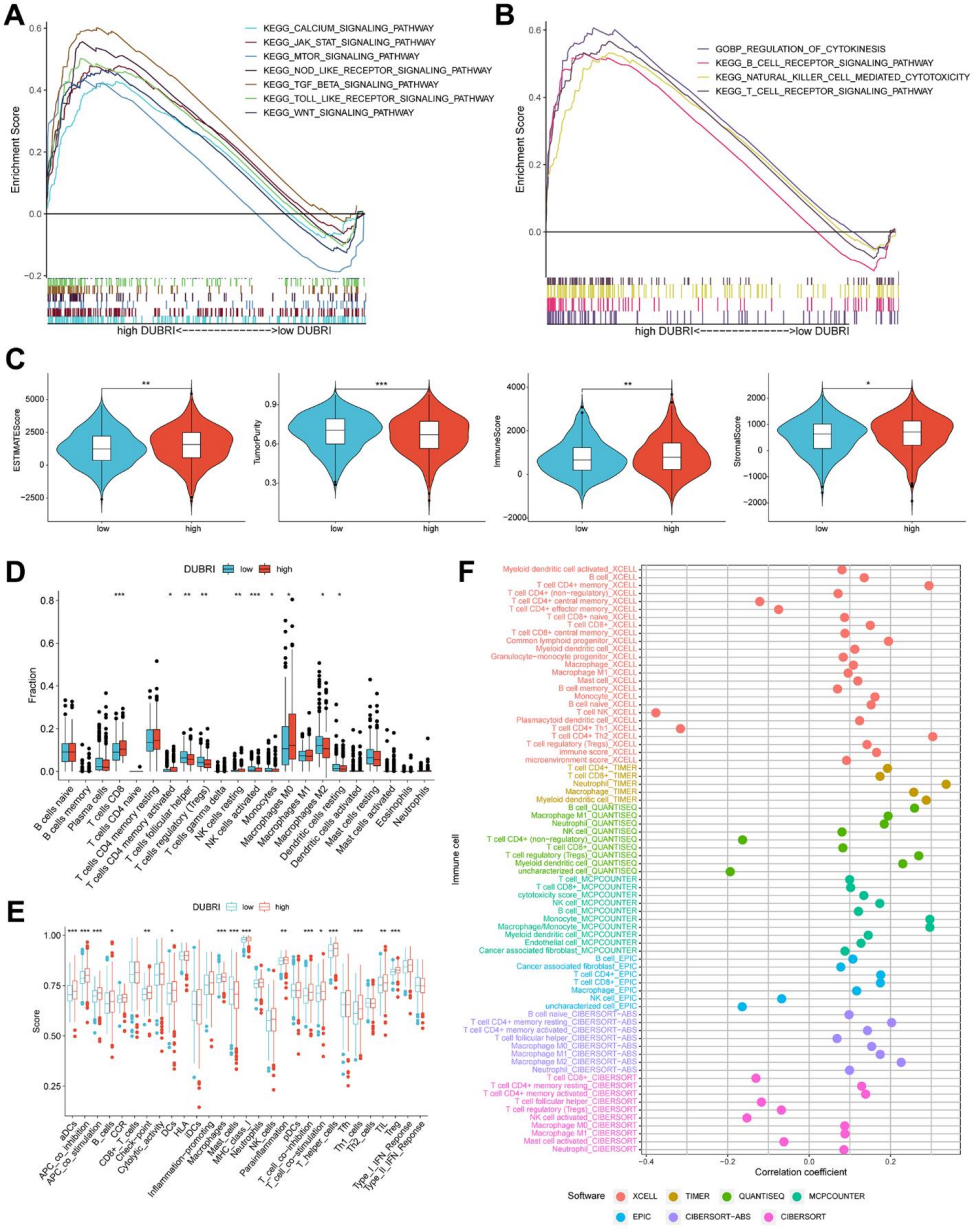
**Assessment of the tumor microenvironment based on the deubiquitinating enzyme-related index.** (**A**, **B**) GSEA analysis was conducted on patients belonging to the high DUBRI group. (**C**) Variations in tumor purity, ESTIMATEScore, immunity score, and stroma score observed between high and low DUBRI groups. (**D**) The CIBERSORT technique is used to evaluate the variations in immune cell populations between individuals classified as either high or low DUBRI group. (**E**) The ssGSEA method evaluates disparities in immune cells and immunological function across patients categorized into the high/low DUBRI group. (**F**) The connection between DUBRI and immune cell infiltration was analyzed using XCELL, TIMER, QUANTISEQ, MCPCOUNTER, EPIC, CIBERSORT-ABS, and CIBERSORT software.

We used GSEA analysis on breast cancer sample data to examine the pathways in the high/low DUBRI group. The findings indicated that the WNT signaling pathway, CALCIUM, JAK_STAT, MTOR, nod like receptor, tgf beta, and toll like receptor were significantly enriched in the high DUBRI group (Figure 2A). The high DUBRI group were significantly enriched in the TME pathways of REGULATION_OF_CYTOKINESIS, B_CELL_RECEPTOR, NATURAL_KILLER_CELL, and T_CELL_RECEPTOR, which suggests that our high DUBRI group is closely related to the TMB is closely related (Figure 2B).

We discovered that the high DUBRI group had greater stroma, immunity, and ESTIMATE scores but poorer tumor purity using the ESTIMATE technique (Figure 2C). The CIBERSORT algorithm revealed that the high DUBRI group had significantly higher levels of immunostimulated CD8 T cells than the low DUBRI group, while the high DUBRI group had significantly lower levels of immunosuppressed M2-type macrophages. These findings suggested that the high DUBRI group had high immune infiltration characteristics (Figure 2D). The findings of the ssGSEA algorithm demonstrated that patients in the high DUBRI group had greater immune cell infiltration and immune-related functions than those in the low DUBRI group. For example, the high DUBRI group’s level of immunological checkpoints was much higher than that of the low DUBRI group (Figure 2E). We also found a correlation between DUBRI and different immune cell infiltration by different software (Figure 2F).

### Efficacy of DUBRI in predicting immunotherapy effects

**Figure 3:**
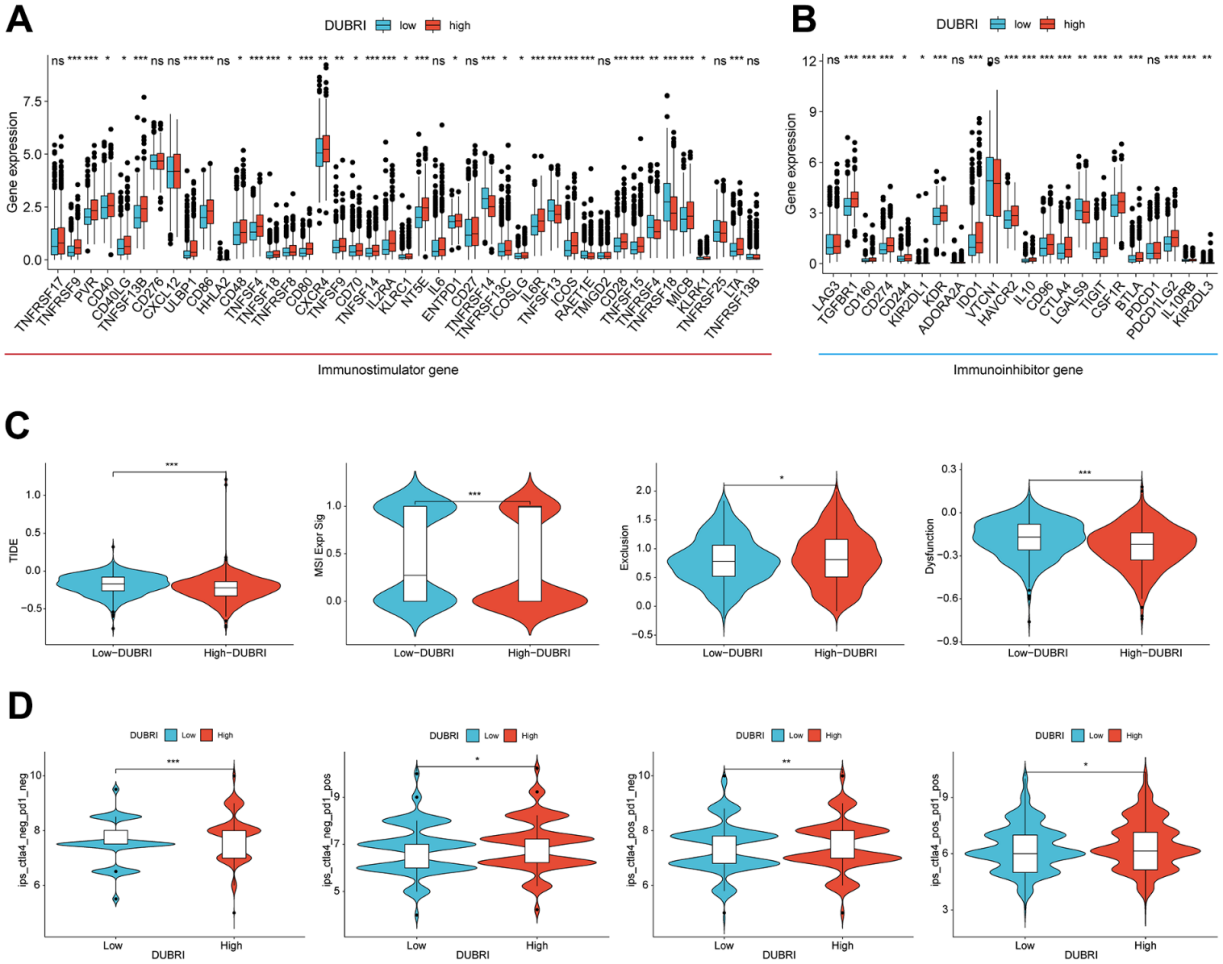
**Efficacy of DUBRI in predicting immunotherapy outcome.** (**A**, **B**) Differences in expression levels of common immune checkpoints between high/low DUBRI groups. (**C**) Difference between TIDE score, Dysfunction, MSI and Exclusion between high/DUBRI and low DUBRI groups. (**D**) Differences in IPS scores between high/low DUBRI groups.

We compared the expression levels of common immune checkpoints (immune-stimulating and immune-suppressing genes) between the high and low DUBRI groups in order to further investigate the relationship between DUBRI and the immune microenvironment. The findings demonstrated that the patients in the high DUBRI group had significantly higher expression levels of most immune checkpoints than those in the low DUBRI group (Figure 3A, 3B). By the above results of ESTIMATE analysis, the high DUBRI group had high immune infiltration characteristics.

Patients with lower TIDE scores had longer survival times and were more likely to benefit from immunotherapy [9]. Compared to patients in the low DUBRI group, patients in the high DUBRI group had greater MSI and Exclusion, lower TIDE scores, and Dysfunction, indicating that their immune checkpoint blockade treatment was more effective (Figure 3C). We subsequently investigated the correlation between the DUBRI group and the Immunophenotypic Score (IPS), which is a tool used to predict how patients will respond to ICB treatments such as anti-PD1 and anti-CTLA4. By assessing immunogenicity, we discovered that patients in the high-DUBRI group had higher IPS scores. This indicates that individuals in the high-DUBRI group may have a more favorable response to immune therapy (Figure 3D). In addition, we downloaded and analyzed DUBRI in the external validation data GSE173839 and GSE194040 data for predicting the effect of immunotherapy, and found that DUBRI was more highly expressed in the immunotherapy-responsive group compared to the immunotherapy-responsive non-responsive group (Supplementary Figure 2).

### DUBRI in predicting drug sensitivity

**Figure 4:**
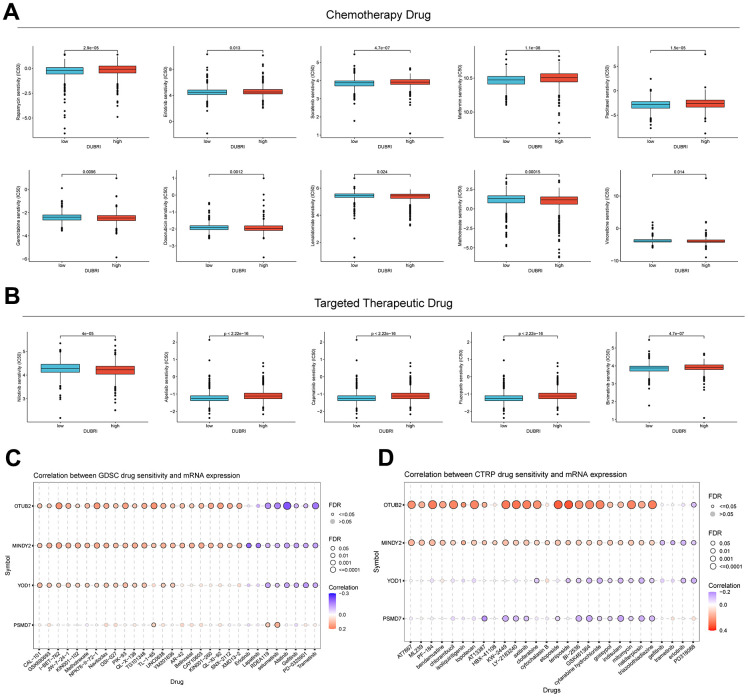
**Value of DUBRI in predicting drug sensitivity.** (**A**) Disparity in the reaction to typical chemotherapy drugs among individuals in the high and low DUBRI groups. (**B**) Variations in the responsiveness to commonly used targeted therapeutic drugs between the high and low DUBRI groups. (**C**, **D**) The association between drug sensitivity and mRNA expression of the four genes that make up PCDI was studied using the GDSC database and CTRP database.

In order to investigate the correlation between DUBRI and medication sensitivity, we determined the IC50 values of commonly used chemotherapeutic and targeted therapeutic drugs in breast cancer samples. We then compared these values across different DUBRI subgroups. We discovered notable variations in the chemotherapeutic and targeted therapeutic drugs used for breast cancer treatment across the high/low DUBRI groups. The IC50 values for Gemcitabine were much lower in the high DUBRI group compared to the low DUBRI group, indicating that patients in the high DUBRI group would exhibit a more favorable response to Gemcitabine-based chemotherapy. (Figure 4A, 4B). A positive correlation suggested that gene expression was linked to drug resistance, whereas a negative correlation indicated that gene expression was linked to drug sensitivity. The findings demonstrated a significant correlation between the expression of OTUB2 and MINDY2 genes with the majority of medication sensitivities (Figure 4C, 4D).

### Molecular docking of the target protein OTUB2

**Figure 5:**
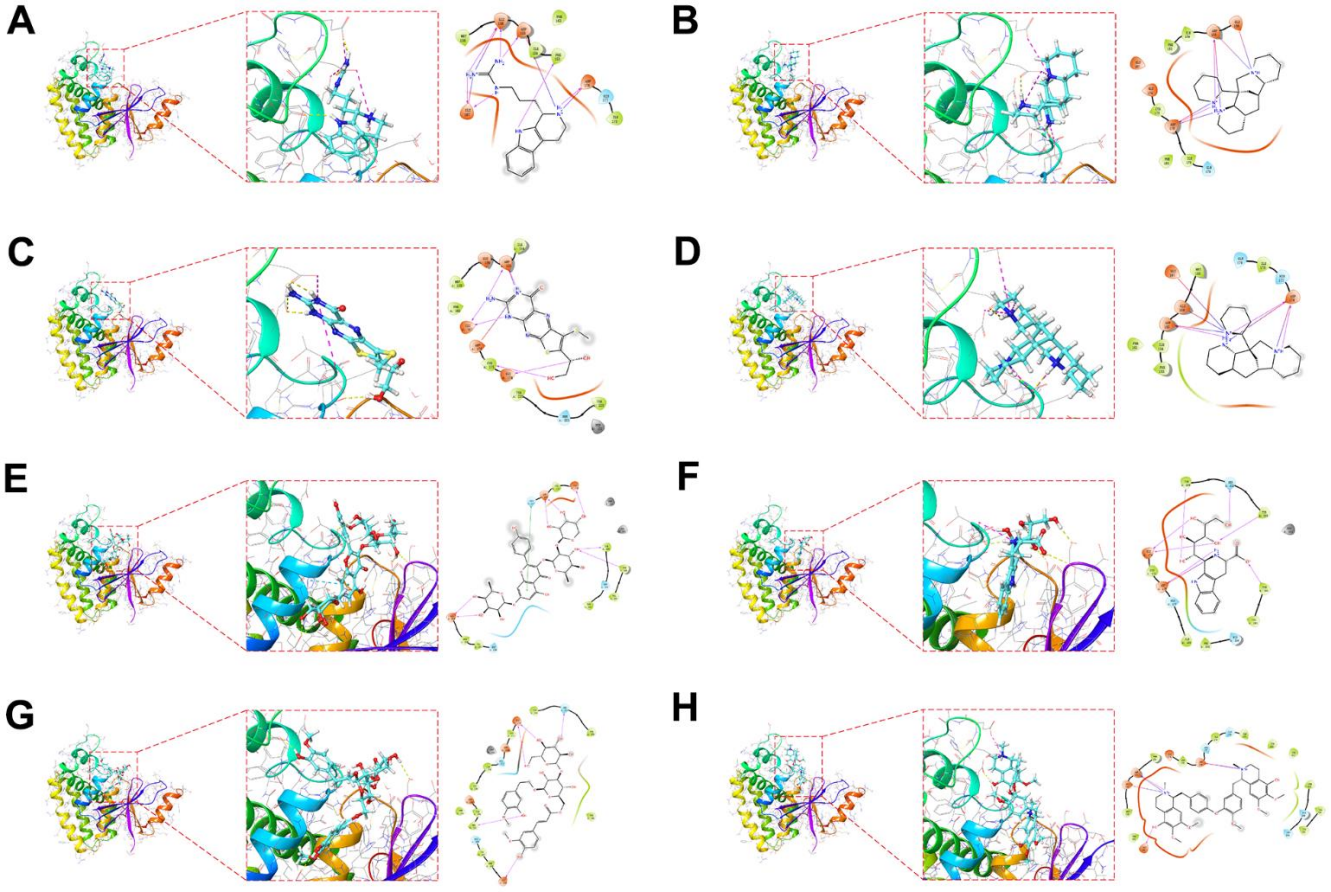
**Molecular docking pose.** Utilizing molecular docking to screen potential small compounds for target proteins. Figure shows the OTUB2 active pocket with Trypargine (**A**), Ormosanine (**B**), Urothion (**C**), Piptanthine (**D**), Sagittatin A (**E**), Tetrahydropentoxyline (**F**), Scroside B (**G**) and Thalirugidine (**H**) in docking position.

We obtained the protein structure of OTUB2 from the PDB database for molecular docking with small molecule compounds. The top eight small molecules (Trypargine, Ormosanine, Urothion, Piptanthine, Sagittatin A, Tetrahydropentoxyline, Scroside B, and Thalirugidine) with the highest binding affinity to the binding pocket of OTUB2 were shown (Figure 5A–5H). For example, Tetrahydropentoxyline forms hydrogen bonds with OTUB2 amino acid residues Glu-174, Asp-176, Tyr-195, Tyr-225, Ser-223, and Tyr-220, with Glu-174, Asp-176, Tyr-195, Tyr-225, and Ser-223 acting as the hydrogen bond acceptor and Tyr-220 as hydrogen bond donor. Scroside B forms hydrogen bonds with OTUB2 amino acid residues Glu-210, Phe-153, Glu-174, and Ser-223, wherein Glu-210, Phe-153, Glu-174, and Ser-223 act as hydrogen bond acceptors.

### Knockdown of OTUB2 inhibits breast cancer cell proliferation and migration

**Figure 6:**
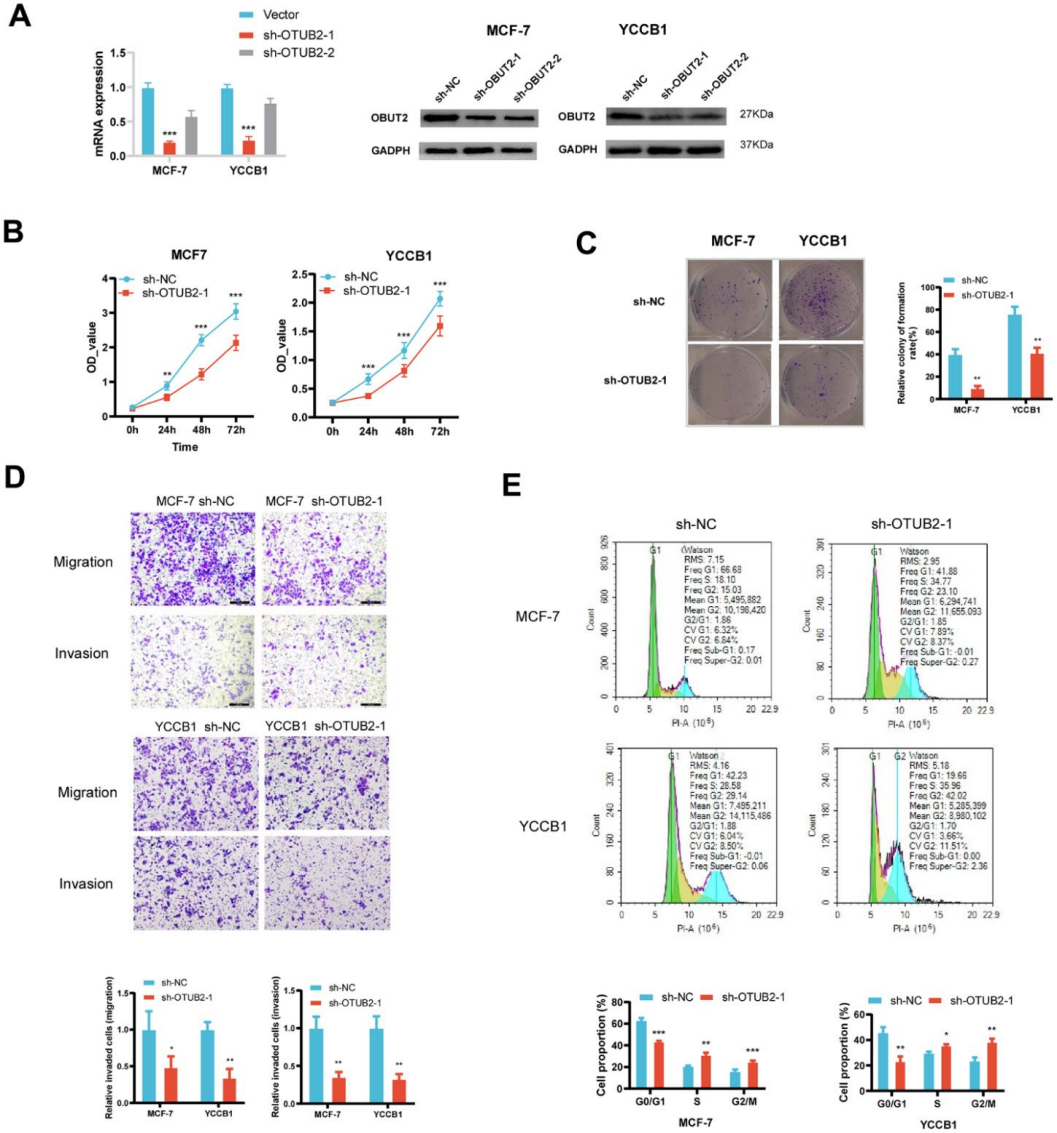
**Construction of the OTUB2 knockdown cell and the functional experiment.** (**A**) OTUB2 was successfully knocked down in MCF-7 and YCCB1 cells using lentiviral transfection method. (**B**) The CCK8 experiment showed that the number of OTUB2 knockdown cells decreased compared to sh-NC cells at various time points. (**C**) The clone formation experiment suggests that OTUB2 knockdown cells may have weaker formation ability than sh-NC cells. (**D**) Transwell experiment observed the changes in migration and invasion ability of OTUB2 knockdown cells compared to sh-NC cells. (**E**) Cell cycle distribution of OTUB2 knockdown cells and sh-NC cells were detected by flow cytometry.

OTUB2 was knocked down by shRNA in MCF-7 and YCCB1 cells *in vitro* (Figure 6A). In the CCK8 experiment, the cell number was decreased in sh-OTUB2-1 group compared to the sh-NC group at 24h, 48h and 72h, indicating that the cell proliferation ability decreased after OTUB2 silencing (Figure 6B). In the clone formation experiment, knockdown of OTUB2 significantly reduced the clone formation ability in MCF-7 and YCCB1 cells (Figure 6C). In Transwell experiment, the OTUB2 silenced group showed a decrease in the number of cells penetrating to the lower chamber compared to the sh-NC group, which manifested OTUB2 silencing lead to a decrease in migration and invasion ability (Figure 6D). Finally, cell cycle detection revealed that the proportion of cells in G2/M and S phases was significantly higher in sh-OTUB2-1 group than that in sh-NC group, suggesting that cells were arrested in G2/M and S phases after OTUB2 silencing (Figure 6E).

## DISCUSSION

More and more studies are now showing that targeting tumor deubiquitinating enzymes is an important anticancer therapeutic strategy. Deubiquitinating enzymes can not only regulate druggable proteins, but also modulate the levels of proteins that are insensitive to conventional targeted therapies or difficult to intervene with small molecules, making them a class of highly promising drug targets. Therefore, exploring the mechanism and function of deubiquitinating enzymes will provide some important insights [4, 12] for future cancer therapy. In this study, Deubiquitinating enzyme related index (DUBRI) was constructed based on five deubiquitinating enzyme genes (OTUB2, USP41, MINDY2, YOD1 and PSMD7). Our research has shown that DUBRI may serve as a reliable indicator for classifying breast cancer and accurately predicting the prognosis and response to immunotherapy in breast cancer patients. This finding has been confirmed by independent datasets. Furthermore, we have discovered natural small compounds that can specifically bind to the central target protein of deubiquitinating enzymes using molecular docking. Ultimately, the inverse relationship between OTUB2 and CD8 T cells was validated using immunohistochemistry.

The occurrence and development of a tumor are intricately linked to its capacity to modify the tumor microenvironment in order to avoid immune monitoring [13–17]. In order to elucidate the connection between DUBRI and the tumor microenvironment in breast cancer, we conducted an analysis and found that patients in the high DUBRI group exhibited a strong correlation with elevated levels of immune cell infiltration in the TMB. This was determined through the utilization of various algorithms including ESTIMATE, GSEA enrichment analysis, ssGSEA, and CIBERSORT. In addition, we compared the expression levels of immune checkpoints between the high/low DUBRI groups and found that patients in the high DUBRI group had significantly higher levels of immune checkpoints than those in the low DUBRI group, suggesting that the tumor cells in the high DUBRI group may have high immune escape potential. We predicted the immunotherapeutic effects of the high DUBRI and low DUBRI groups by anti-PD-1 and anti-CTLA-4 IPS scores and TIDE scoring, and the results showed that patients in the high DUBRI group responded better to immunotherapy. On the other hand, we assessed the relationship between DUBRI and drug sensitivity by IC50 values of common chemotherapeutic and targeted drugs. We found that DUBRI could differentiate breast cancer sensitivity to common chemotherapeutic drugs and targeted drugs. According to the data above, DUBRI may evaluate the effectiveness of immunotherapy and chemotherapeutic medications in patients with breast cancer, which might be very beneficial for those patients’ future care.

The Deubiquitinating enzyme related index (DUBRI) is composed of five deubiquitinating enzyme genes including OTUB2, USP41, MINDY2, YOD1 and PSMD7. OTU Deubiquitinase, Ubiquitin Aldehyde Binding 2 (OTUB2) encodes a deubiquitinase-associated protein that contains an OUT domain and binds Ubal (ubiquitin aldehyde) involved in protein metabolism and deubiquitination. OTUB2 inhibits ubiquitination by interacting with pyruvate kinase M2 (PKM2), blocking the interaction of PKM2 with its ubiquitin E3 ligase, which enhances PKM2 activity and promotes glycolysis. This enhances PKM2 activity and promotes glycolysis, which in turn promotes colorectal cancer progression [18]. Ubiquitin Specific Peptidase 41 (USP41), which is highly expressed in breast cancer, is deubiquitinated by interacting with Snail, which enhances the activity of the Snail protein and promotes breast carcinogenesis EMT [19]. MINDY Lysine 48 Deubiquitinase 2 (MINDY2) interacts with and deubiquitinates ACTN4, stabilizes ACTN4, and activates the PI3K/AKT/mTOR signaling pathway to promote pancreatic cancer proliferation and metastasis [20]. Yod1 Deubiquitinase (YOD1) is a protein coding gene that is expressed in tumor cells [12, 21, 22]. Proteasome 26S Subunit, Non-ATPase 7 (PSMD7) is highly expressed in gastric cancer. Stabilizes RAD23B by deubiquitination, which in turn promotes proliferation, invasion and cisplatin resistance in gastric cancer [23].

## CONCLUSIONS

In summary, our comprehensive analysis of multiple aspects of breast cancer based on DUBRI constructed from deubiquitinating enzyme genes. We discovered that DUBRI is very proficient in forecasting the prognosis and immunotherapy response of breast cancer patients. This has been confirmed by external datasets. In addition, we have discovered novel prognostic and therapeutic biomarkers for breast cancer, as well as specific small molecule medicines, by focusing on deubiquitinating enzymes. These findings provide valuable insights for the development of precise treatments for breast cancer in the future. During a time when immunotherapy shows significant potential for treating cancer, DUBRI offers valuable guidance for the clinical diagnosis and personalized, complete treatment of breast cancer.

## Supplementary Material

Supplementary Figures

Supplementary Tables

## References

[1] Sung H, Ferlay J, Siegel RL, Laversanne M, Soerjomataram I, Jemal A, Bray F. Global Cancer Statistics 2020: GLOBOCAN Estimates of Incidence and Mortality Worldwide for 36 Cancers in 185 Countries. CA Cancer J Clin. 2021; 71:209–49. 10.3322/caac.21660 33538338

[2] Xu T, Zhang SM, Wu HM, Wen XM, Qiu DQ, Yang YY, Wang LZ, Zhu WB, He LS, Li JJ. Prognostic significance of prognostic nutritional index and systemic immune-inflammation index in patients after curative breast cancer resection: a retrospective cohort study. BMC Cancer. 2022; 22:1128. 10.1186/s12885-022-10218-x 36329394 PMC9632068

[3] Bianchini G, Balko JM, Mayer IA, Sanders ME, Gianni L. Triple-negative breast cancer: challenges and opportunities of a heterogeneous disease. Nat Rev Clin Oncol. 2016; 13:674–90. 10.1038/nrclinonc.2016.66 27184417 PMC5461122

[4] Harrigan JA, Jacq X, Martin NM, Jackson SP. Deubiquitylating enzymes and drug discovery: emerging opportunities. Nat Rev Drug Discov. 2018; 17:57–78. 10.1038/nrd.2017.152 28959952 PMC7097658

[5] Zou Q, Jin J, Hu H, Li HS, Romano S, Xiao Y, Nakaya M, Zhou X, Cheng X, Yang P, Lozano G, Zhu C, Watowich SS, et al. USP15 stabilizes MDM2 to mediate cancer-cell survival and inhibit antitumor T cell responses. Nat Immunol. 2014; 15:562–70. 10.1038/ni.2885 24777531 PMC4032322

[6] Shah P, Kendall F, Khozin S, Goosen R, Hu J, Laramie J, Ringel M, Schork N. Artificial intelligence and machine learning in clinical development: a translational perspective. NPJ Digit Med. 2019; 2:69. 10.1038/s41746-019-0148-3 31372505 PMC6659652

[7] Liu J, Lichtenberg T, Hoadley KA, Poisson LM, Lazar AJ, Cherniack AD, Kovatich AJ, Benz CC, Levine DA, Lee AV, Omberg L, Wolf DM, Shriver CD, et al, and Cancer Genome Atlas Research Network. An Integrated TCGA Pan-Cancer Clinical Data Resource to Drive High-Quality Survival Outcome Analytics. Cell. 2018; 173:400–16.e11. 10.1016/j.cell.2018.02.052 29625055 PMC6066282

[8] Yang W, Soares J, Greninger P, Edelman EJ, Lightfoot H, Forbes S, Bindal N, Beare D, Smith JA, Thompson IR, Ramaswamy S, Futreal PA, Haber DA, et al. Genomics of Drug Sensitivity in Cancer (GDSC): a resource for therapeutic biomarker discovery in cancer cells. Nucleic Acids Res. 2013; 41:D955–61. 10.1093/nar/gks1111 23180760 PMC3531057

[9] Jiang P, Gu S, Pan D, Fu J, Sahu A, Hu X, Li Z, Traugh N, Bu X, Li B, Liu J, Freeman GJ, Brown MA, et al. Signatures of T cell dysfunction and exclusion predict cancer immunotherapy response. Nat Med. 2018; 24:1550–8. 10.1038/s41591-018-0136-1 30127393 PMC6487502

[10] Charoentong P, Finotello F, Angelova M, Mayer C, Efremova M, Rieder D, Hackl H, Trajanoski Z. Pan-cancer Immunogenomic Analyses Reveal Genotype-Immunophenotype Relationships and Predictors of Response to Checkpoint Blockade. Cell Rep. 2017; 18:248–62. 10.1016/j.celrep.2016.12.019 28052254

[11] Ritchie ME, Phipson B, Wu D, Hu Y, Law CW, Shi W, Smyth GK. limma powers differential expression analyses for RNA-sequencing and microarray studies. Nucleic Acids Res. 2015; 43:e47. 10.1093/nar/gkv007 25605792 PMC4402510

[12] Wu Y, Duan Y, Han W, Cao J, Ye B, Chen P, Li H, Wang Y, Liu J, Fang Y, Yue K, Wu Y, Wang X, Jing C. Deubiquitinase YOD1 suppresses tumor progression by stabilizing E3 ligase TRIM33 in head and neck squamous cell carcinoma. Cell Death Dis. 2023; 14:517. 10.1038/s41419-023-06035-0 37573347 PMC10423255

[13] Argilés JM, López-Soriano FJ, Stemmler B, Busquets S. Cancer-associated cachexia - understanding the tumour macroenvironment and microenvironment to improve management. Nat Rev Clin Oncol. 2023; 20:250–64. 10.1038/s41571-023-00734-5 36806788

[14] Xu Y, Hua J, Que H, Zeng T, Li Q, Deng J, Xie J. Identification of PANoptosis-related signature reveals immune infiltration characteristics and immunotherapy responses for renal cell carcinoma. BMC Cancer. 2024; 24:292. 10.1186/s12885-024-12067-2 38439022 PMC10913266

[15] Xu Y, Li Q, Lin H. Bioinformatics analysis of CMTM family in pan-cancer and preliminary exploration of CMTM6 in bladder cancer. Cell Signal. 2024; 115:111012. 10.1016/j.cellsig.2023.111012 38113979

[16] Jiang F, Xu Y, Jiang Z, Hu B, Lv Q, Wang Z. Deciphering the immunological and prognostic features of hepatocellular carcinoma through ADP-ribosylation-related genes analysis and identify potential therapeutic target ARFIP2. Cell Signal. 2024; 117:111073. 10.1016/j.cellsig.2024.111073 38302034

[17] Xu Y, Xia Z, Sun X, Wei B, Fu Y, Shi D, Zhu Y. Identification of a glutamine metabolism reprogramming signature for predicting prognosis, immunotherapy efficacy, and drug candidates in bladder cancer. Front Immunol. 2023; 14:1111319. 10.3389/fimmu.2023.1111319 36911676 PMC9995899

[18] Yu S, Zang W, Qiu Y, Liao L, Zheng X. Deubiquitinase OTUB2 exacerbates the progression of colorectal cancer by promoting PKM2 activity and glycolysis. Oncogene. 2022; 41:46–56. 10.1038/s41388-021-02071-2 34671086

[19] Yoon JY, Seo SU, Woo SM, Kwon TK. USP41 Enhances Epithelial-Mesenchymal Transition of Breast Cancer Cells through Snail Stabilization. Int J Mol Sci. 2023; 24:1693. 10.3390/ijms24021693 36675208 PMC9863231

[20] Liu P, Liu S, Zhu C, Li Y, Li Y, Fei X, Hou J, Wang X, Pan Y. The deubiquitinating enzyme MINDY2 promotes pancreatic cancer proliferation and metastasis by stabilizing ACTN4 expression and activating the PI3K/AKT/mTOR signaling pathway. Front Oncol. 2023; 13:1169833. 10.3389/fonc.2023.1169833 37207150 PMC10189038

[21] Han Z, Jia Q, Zhang J, Chen M, Wang L, Tong K, He W, Zhang Y, Zhu W, Qin J, Wang T, Liu T, Ma Y, et al. Deubiquitylase YOD1 regulates CDK1 stability and drives triple-negative breast cancer tumorigenesis. J Exp Clin Cancer Res. 2023; 42:228. 10.1186/s13046-023-02781-3 37667382 PMC10478497

[22] Shao X, Chen Y, Wang W, Du W, Zhang X, Cai M, Bing S, Cao J, Xu X, Yang B, He Q, Ying M. Blockade of deubiquitinase YOD1 degrades oncogenic PML/RAR α and eradicates acute promyelocytic leukemia cells. Acta Pharm Sin B. 2022; 12:1856–70. 10.1016/j.apsb.2021.10.020 35847510 PMC9279643

[23] Wang J, Liu R, Mo H, Xiao X, Xu Q, Zhao W. Deubiquitinase PSMD7 promotes the proliferation, invasion, and cisplatin resistance of gastric cancer cells by stabilizing RAD23B. Int J Biol Sci. 2021; 17:3331–42. 10.7150/ijbs.61128 34512150 PMC8416741

