# Supplementary material for: The deubiquitinating enzymes-related signature predicts the prognosis and immunotherapy response in breast cancer
Source: Aging (Albany NY). 2024 Jul 9;16(15):11553–67. doi: 10.18632/aging.206010 (PMC11346791; doi:10.18632/aging.206010)
Supplement: Supplementary Figures [file aging-16-206010-s001.pdf]

SUPPLEMENTARY FIGURES

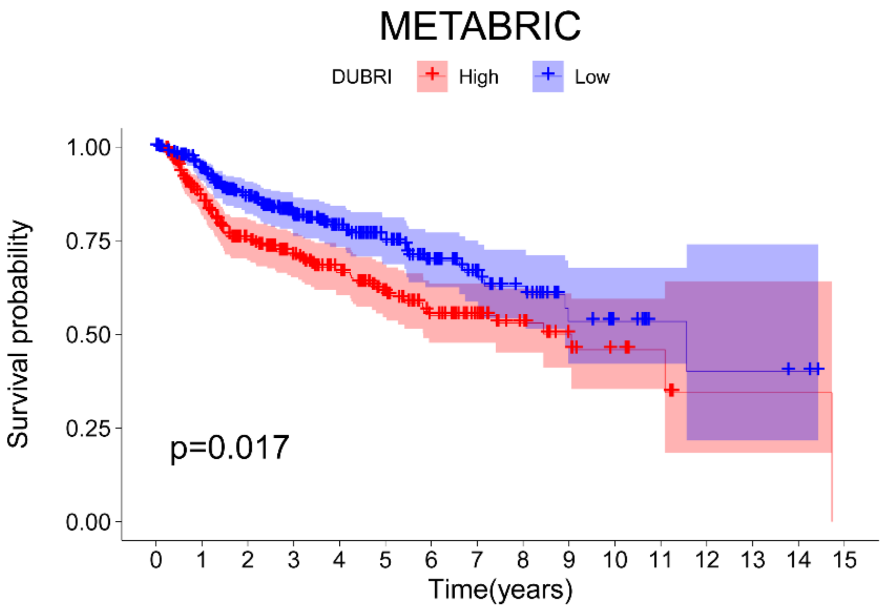

Supplementary Figure 1. Kaplan-Meier survival curves comparing high/low DUBRI for METABRIC dataset.

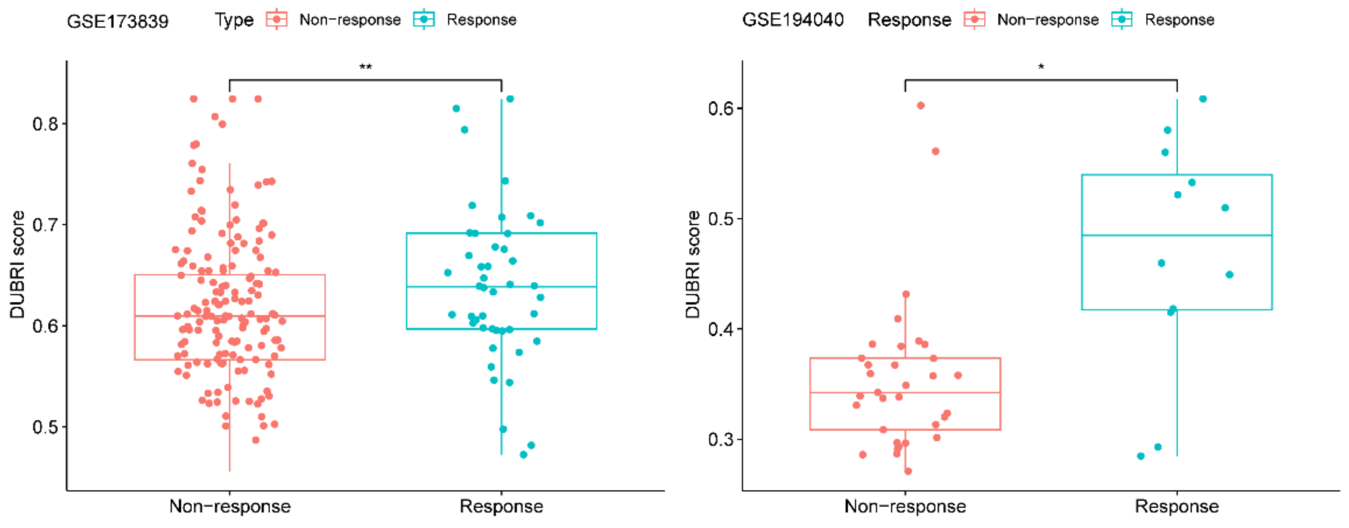

Supplementary Figure 2. External immunotherapy datasets GSE173839 and GSE194040 validate DUBRI for predicting immunotherapy response.
