# Supplementary material for: The deubiquitinating enzymes-related signature predicts the prognosis and immunotherapy response in breast cancer
Source: Aging (Albany NY). 2024 Jul 9;16(15):11553–67. doi: 10.18632/aging.206010 (PMC11346791; doi:10.18632/aging.206010)
Supplement: Supplementary Tables [file aging-16-206010-s002.pdf]

SUPPLEMENTARY TABLES

Supplementary Table 1. Deubiquitinating enzyme genes.

CYLD	USP26
USPL1	USP29
PAN2	USP37
USP53	USP22
USP54	MINDY1
USP1	MINDY2
USP10	MINDY3
USP39	MINDY4
USP11	ZUP1
USP15	OTUD1
USP4	OTUD4
USP8	OTUD4P1
USP19	OTULIN
USP2	OTULINL
USP21	OTUD5
USP50	OTUD3
USP20	OTUD6A
USP33	OTUD6B
USP12	YOD1
USP46	OTUB1
USP27X	OTUB2
USP51	OTUD7A
USP3	OTUD7B
USP44	TNFAIP3
USP49	ZRANB1
USP35	VCPIP1
USP38	BRCC3
USP18	COPS5
USP41	PSMD14
USP24	MPND
USP9X	MYSM1
USP9Y	STAMBP
USP34	STAMBPL1
USP47	PRPF8
USP7	COPS6
USP48	EIF3F
USP40	PSMD7
USP14	EIF3H
USP30	ATXN3
USP16	ATXN3L
USP45	JOSD1
USP31	JOSD2
USP43	BAP1
USP32	UCHL5
USP6	UCHL1
USP13	UCHL3
USP5	
USP25	
USP28	

Supplementary Table 2. The clinical characteristics of breast cancer samples in the TCGA training set, GSE20685, and GSE88770 validation sets.

Characteristics	TCGA cohort	GSE20685 cohort	GSE88770 cohort
**n**	1049	327	117
**Age, n (%)**			
≤65	756 (72.1%)	305 (93.3%)	/
>65	293 (27.9%)	22 (6.7%)	/
**WHO stage, n (%)**			
I	183 (17.5%)	/	/
II	592 (56.4%)		
III	233 (22.2%)		
IV	18 (1.7%)		
X	12 (1.1%)		
Unknown	11 (1.1%)		
**T stage, n (%)**			
T1	280 (26.7%)	101 (30.9%)	/
T2	599 (57.1%)	188 (57.5%)	
T3	132 (12.6%)	26 (7.9%)	
T4	35 (3.3%)	12 (3.7%)	
TX	3 (0.3%)		
**N stage, n (%)**			
N0	491 (46.8%)	137 (41.9%)	/
N1	356 (34.0%)	87 (26.6%)	
N2	110 (10.5%)	63 (19.3%)	
N3	75 (7.1%)	40 (12.2%)	
NX	17 (1.6%)		
**M stage, n (%)**			
M0	867 (82.7%)	319 (97.6%)	/
M1	20 (1.9%)	8 (2.4%)	
MX	162 (15.4%)		
**Survival status, n (%)**			
Alive	898 (85.6%)	244 (74.6%)	/
Dead	151 (14.4%)	83 (25.4%)	/
